# Synthesis and Characterization of CNC/CNF/rGO Composite Films for Advanced Functional Applications

**DOI:** 10.3390/mi17030387

**Published:** 2026-03-23

**Authors:** Ghazaleh Ramezani, Ion Stiharu, Theo G. M. van de Ven, Hossein Ramezani, Vahe Nerguizian

**Affiliations:** 1 Department of Mechanical and Industrial Engineering, Concordia University, Montreal, QC H3G 1M8, Canada; ghazaleh.ramezani@mail.concordia.ca; 2 Department of Chemistry, McGill University, Montreal, QC H4A 3J1, Canada; theo.vandeven@mcgill.ca; 3 Department of Mechanical Engineering, Clemson University, Clemson, SC 29634, USA; hrameza@clemson.edu; 4 Département de Génie Électrique, École de Technologie Supérieure, Montreal, QC H3C 1K3, Canada; vahe.nerguizian@etsmtl.ca

**Keywords:** cellulose nanocrystals, cellulose nanofibrils, reduced graphene oxide, composite films, mechanical properties, electrical conductivity, dielectric spectroscopy

## Abstract

Developing advanced functional materials requires the synergistic integration of nanoscale reinforcements with tailored properties. In this work, composite films of cellulose nanocrystals (CNCs), cellulose nanofibrils (CNFs), and reduced graphene oxide (rGO) were synthesized using a combination of solution casting, high shear homogenization, vacuum filtration, and environmentally friendly chemical reduction. The resulting CNC/CNF/rGO films exhibited a robust hierarchical structure with strong interfacial interactions, enabling exceptional mechanical properties, specifically a tensile strength of 215 MPa and a Young’s modulus of 18 GPa, alongside a continuous conductive network confirmed by frequency-independent electrical conductivity up to 30 kHz. Comprehensive dielectric characterization revealed frequency-dependent permittivity and low dielectric loss, aligning with Maxwell–Wagner theoretical predictions for heterogeneous composites. The composites also demonstrated thermal stability, with electrical conductivity increasing monotonically from 0 °C to 200 °C. These findings highlighted the CNC/CNF/rGO films’ suitability for applications in flexible electronics, electromagnetic shielding, packaging, and high-performance structural materials. Future optimization and modeling approaches, including fractional calculus, are recommended to further enhance multifunctionality and exploit the unique synergistic interactions intrinsic to nanocellulose–graphene oxide platforms.

## 1. Introduction

GO, a two-dimensional carbon nanomaterial obtained through the oxidation of graphite, exhibits a distinctive structure composed of a single layer of carbon atoms functionalized with oxygen-containing groups, including hydroxyl, epoxide, and carboxyl moieties [1]. Among the most widely employed synthesis routes, the modified Hummers’ method—which involves the oxidation of graphite using potassium permanganate in concentrated sulfuric acid—remains the predominant approach due to its scalability and reproducibility [2]. These oxygen-containing functionalities endow GO with strong hydrophilicity and excellent dispersibility in aqueous systems. When GO undergoes chemical reduction (e.g., using hydrazine, ascorbic acid, or hydroiodic acid) or thermal annealing, it transforms into rGO, which exhibits greatly enhanced electrical conductivity (up to ~10^3^ S/m) and a partially restored sp^2^ carbon network, all while retaining functional surface groups suitable for composite integration [3,4,5,6]. Nanocellulose, which includes both cellulose nanocrystals (CNCs) and cellulose nanofibrils (CNFs), represents a versatile class of bio-based nanomaterials with well-documented outstanding structural and functional attributes [1,7]. CNCs are typically short, rod-shaped particles (length: 100–500 nm; diameter: 5–10 nm) derived from cellulose by acid hydrolysis, exhibiting a crystallinity index of 54–88%, a theoretical axial elastic modulus of approximately 150 GPa, and a specific surface area of 150–250 m^2^/g [8,9]. In contrast, CNFs consist of long, flexible fibrils (width: 5–20 nm; length: 1–4 µm; aspect ratio > 100) typically produced by mechanical refining or TEMPO-mediated oxidation of cellulose pulp, with a surface carboxylate content of 0.5–1.5 mmol/g depending on the pretreatment [10,11]. Both CNCs and CNFs exhibit a density of approximately 1.5–1.6 g/cm^3^ and retain thermal stability up to 200–300 °C depending on their surface chemistry, which makes them particularly attractive for composite reinforcement and functional material systems [10,11,12,13]. When used together, these two nanocellulosic forms can complement each other’s structural roles, giving rise to hybrid materials with potentially synergistic mechanical and functional properties [14]. The motivation for integrating GO or rGO with nanocellulose arises from the complementary nature of their properties and the opportunity to achieve synergistic effects when incorporated into composite systems. GO and rGO impart quantifiably superior properties to composite materials: rGO electrical conductivity ranges from 10^2^ to 10^3^ S/m depending on reduction degree and defect density [15]; the in-plane thermal conductivity of rGO films is reported at 5–1500 W/m·K [16]; and the Young’s modulus of single-layer graphene is ~1 TPa, with rGO retaining ~0.25 TPa after chemical reduction due to structural defects [17]. Nanocellulose, in turn, contributes reinforcement efficiency factors of 50–100% improvement in tensile strength over unreinforced polymer matrices [18], as well as biodegradability and enhanced filler dispersion arising from its high surface charge and hydroxyl functionality [19]. Together, these complementary property profiles enable the design of multifunctional composites with precisely tunable characteristics for water purification, energy storage systems, and biomedical applications [20].

Recent progress in GO/rGO–nanocellulose composites has opened new possibilities across multiple areas of research and technology [21,22]. In water purification, for instance, scientists have created highly efficient adsorbent materials by integrating GO with cellulose nanocrystals. The resulting structures form hierarchically porous and mechanically resilient sponges that demonstrate an outstanding ability to capture and retain a wide range of water contaminants [8,9]. These hybrid composites display notably stronger mechanical integrity, a larger effective surface area, and superior adsorption performance when compared with either of their individual constituents [23,24]. In the area of energy storage, GO/rGO–nanocellulose composites have emerged as high-performance electrode materials for both supercapacitors and lithium-ion batteries. For instance, self-supported CNF/rGO supercapacitor electrodes have demonstrated a specific capacitance of 158 F/g at 0.5 A/g, with a capacitance retention of approximately 90% after 5000 charge–discharge cycles. The electrical conductivity of rGO-rich composite films in such systems typically falls in the range of 10–100 S/m, sufficient for percolative charge transport [25,26]. Regarding thermal resilience, nanocellulose-based separators for lithium-ion batteries exhibit thermal shrinkage below 5% at 120 °C, compared to over 40% for conventional polyolefin separators, substantially improving battery safety under thermal abuse conditions [27]. Importantly, under elevated temperatures, such as those encountered during electric vehicle (EV) battery operation, nanocellulose-based materials used in separators and composite structures have demonstrated superior thermal stability and lower shrinkage than conventional polyolefin separators. These characteristics contribute significantly to improving the overall safety and maintaining the dimensional integrity of advanced battery systems [28]. Studies have shown that integrating nanocellulose with ceramic fillers, crosslinked polymers, or high-temperature surface treatments enables these materials to withstand thermal runaway, all while preserving their electrochemical performance at temperatures approaching, and in some cases exceeding, 120 °C. This combination of properties positions GO/rGO–nanocellulose composites as strong contenders for next-generation battery systems where both exceptional thermal endurance and mechanical strength are essential [29,30].

While numerous studies have examined binary GO/rGO–nanocellulose composites, the present work distinguishes itself in three key respects. First, it employs a ternary nanocellulosic architecture combining both CNCs and CNFs simultaneously with rGO, exploiting the complementary geometries of rod-like nanocrystals and long, flexible nanofibrils to build a hierarchically reinforced network. Second, the chemical reduction in GO is performed in situ within the cast composite film using L-ascorbic acid, an environmentally benign reducing agent, rather than pre-reducing GO prior to film assembly, a distinction that preserves the interfacial interactions established during casting. Third, this study provides comprehensive multiscale characterization spanning morphology, particle size distribution, tensile mechanics, broadband electrical conductivity (1 Hz–1 MHz), dielectric permittivity, dielectric loss, and temperature-dependent conductivity (0–200 °C) within a single study and interprets these results within a unified Maxwell–Wagner effective medium framework.

Despite considerable progress, a critical gap remains in the literature, as few studies have systematically combined both CNCs and CNFs with rGO in a single composite film while simultaneously employing a green reduction strategy and conducting comprehensive multiscale characterization encompassing mechanical, electrical, and broadband dielectric properties. Moreover, the applicability of effective medium approximation (EMA) and Maxwell–Wagner models to such ternary nanocomposites has not been thoroughly validated experimentally. The present study addresses these gaps by: (i) synthesizing CNC/CNF/rGO composite films via solution casting and environmentally benign L-ascorbic acid reduction; (ii) characterizing their morphological, mechanical, electrical, and dielectric properties across a broad frequency and temperature range; and (iii) interpreting the results using the Maxwell–Wagner theoretical framework. This integrated experimental and theoretical approach aims to establish CNC/CNF/rGO films as viable multifunctional platforms for advanced applications in flexible electronics, electromagnetic shielding, and structural materials.

Notably, nanocellulose-based separators have demonstrated thermal shrinkage below 5% at 120 °C and superior dimensional stability compared to conventional polyolefin separators, further enhancing the safety and reliability of these composite systems in battery applications [15,16,17,18,29,31,32,33]. The biomedical field has seen notable advances in the application of GO/rGO–nanocellulose composites. By embedding reduced graphene oxide and cellulose nanocrystals into polylactic acid matrices, researchers have engineered scaffolds that have displayed enhanced mechanical strength alongside pronounced antibacterial behavior. Such nanocomposites are emerging as strong candidates for tissue engineering, as they appear to combine the natural biocompatibility of nanocellulose with the intrinsic antimicrobial capacity of rGO in a synergistic manner [33,34]. The combination of GO or rGO with nanocellulose has also opened new pathways in drug-delivery systems, where GO’s high surface area and tunable surface chemistry facilitate precise control over the release kinetics of therapeutic agents [35].

Researchers working on packaging materials have found that mixing GO or rGO with nanocellulose often produces composites that are tougher and more durable, while also limiting moisture transfer and bacterial growth. Films made from these blends usually keep their strength even in humid conditions, which makes them useful for sustainable and long-lasting packaging. The same principle has also been applied to flexible electronics, where the conductivity of rGO and the pliability of nanocellulose can allow the design of thin, biodegradable supports for next-generation devices [34,35].

The effective properties of such heterogeneous nanocomposites can be theoretically estimated using effective medium approximations (EMAs), among which the Maxwell–Wagner model is particularly suited to predict frequency-dependent dielectric and electrical behavior in two-phase systems [23,24,25,26,27]. This theoretical framework has guided experimental design and data interpretation in the present study. This approach has proven especially valuable for heterogeneous systems such as CNC/CNF/rGO composites, as it enables the prediction of bulk electrical, thermal, and dielectric characteristics from the intrinsic properties and volume fractions of individual components.

The dielectric properties of the composite films were modeled using the Maxwell–Wagner effective medium approach, as described in Section 1, to predict the frequency-dependent complex permittivity and compare it with experimental measurements. The self-consistent form of EMAs, which provide a balanced representation of the composite’s multiphase interactions, can be expressed by the following equation [36,37]:

$$\sum_{i}\varphi_{i}\frac{\varepsilon_{i}-\varepsilon_{eff}}{\varepsilon_{i}+2\varepsilon_{eff}}=0$$

where φ*_i_* represents the volume fraction of component; *ε_i_* is the dielectric constant (or any other relevant property) of component *i*; and *ε_eff_* is the effective dielectric constant of the composite [36,37]. This equation provides a framework for understanding how the individual properties of CNCs, CNFs, and rGO contribute to the overall characteristics of the composite film.

In this study, the EMA framework plays a central role because it captures the intricate interactions occurring between nanocellulose components and reduced graphene oxide sheets. Through this model, it becomes possible to anticipate how changes in the relative proportions of CNCs, CNFs, and rGO influence the overall behavior of the composite—insights that can directly inform both experimental design and data interpretation. In addition, the EMA formulation accommodates the influence of microstructural features such as particle geometry and orientation, parameters that have often proved decisive in defining the performance of nanocomposite systems [38,39].

Mastering and implementing the EMA framework in this research not only establishes a solid theoretical basis for experimental investigations but also serves as a strategic tool for fine-tuning the composition and architecture of CNC/CNF/rGO composites toward targeted functionalities. By linking the microscopic arrangement of constituents to their macroscopic performance, this approach helps bridge theory and practice, ultimately enabling the rational design of advanced materials with precisely tailored properties for specialized applications [40].

## 2. Materials and Methods

### 2.1. Materials

Cellulose nanocrystals (CNCs) were obtained from CelluForce Inc. (Montreal, QC, Canada). According to supplier specifications, the CNCs had a length of approximately 150 nm and a diameter of 5–8 nm, with a crystallinity index of ~88% (determined by XRD) and a sulfate half-ester surface group content of ~300 mmol/kg [CelluForce datasheet; [26]. Their FTIR spectrum exhibited characteristic absorption bands at 3340 cm^−1^ (O–H stretching), 1160 cm^−1^ (C–O–C asymmetric stretching), and 1060 cm^−1^ (C–O stretching), consistent with the cellulose I crystal structure [26,41].

Cellulose nanofibrils (CNFs) were also obtained from CelluForce Inc. The CNFs had fibril widths of 5–20 nm and lengths of 1–4 µm, an aspect ratio exceeding 100, and a surface carboxylate content of approximately 0.5–1.0 mmol/g resulting from TEMPO-mediated oxidation [42]. The FTIR bands at 1600 cm^−1^ (carboxylate C=O stretch) and 3300 cm^−1^ (O–H) confirmed the TEMPO-oxidized surface chemistry [43].

Graphene oxide (GO) powder was obtained Graphene oxide (GO) powder was obtained from Graphenea, San Sebastián, Spain, with a manufacturer-specified lateral flake size of 0.5–5 µm and a C/O atomic ratio of 2.1–2.9, as determined by XPS. The GO consisted predominantly of single- to few-layer sheets, with the layer count and structural quality characterized by Raman spectroscopy (D band ~1350 cm^−1^, G band ~1590 cm^−1^, I_D/I_G ≈ 0.9–1.1, consistent with highly oxidized GO) [44]. The FTIR of the as-received GO confirmed the presence of O–H (~3400 cm^−1^), C=O (~1720 cm^−1^), epoxide C–O–C (~1230 cm^−1^), and C=C (~1620 cm^−1^) functional groups [45]. Following L-ascorbic acid reduction, the C/O ratio typically increased to 6–9, and the I_D/I_G ratio rose to 1.1–1.3, reflecting a partial restoration of the sp^2^ carbon network and an increase in defect-free graphitic domains [46,47].

Glycerol (≥99.5%, analytical grade, Sigma-Aldrich) was used as a plasticizer at 5 wt% relative to the total solid content to enhance film flexibility. All other chemicals were of analytical grade (Sigma-Aldrich).

As noted on in situ characterization, the authors have acknowledged that FTIR, XRD, Raman, and XPS analyses of the final CNC/CNF/rGO composite films were not performed in this study and could represent a limitation. The structural and chemical evolution of the composite before and after reduction was therefore inferred from the established literature for these commercial materials and the applied reduction protocol. These characterization experiments are recommended for future work to directly validate the sp^2^ carbon restoration, interfacial bonding, and crystallographic changes in the final composite.

All reported properties corresponded to the optimized 1:1:0.5 CNC:CNF:rGO composition from a single production batch (n = 5 mechanical specimens, n = 3 electrical specimens). Intra-batch repeatability showed ±10% variation in conductivity and ±12% in tensile strength. Inter-batch variability across independent synthesis runs will be systematically investigated in future work.

### 2.2. Synthesis of CNC/CNF/rGO Composite Films

The synthesis and characterization of CNC/CNF/rGO composite films with the optimal ratio of 1:1:0.5 (CNC:CNF:rGO *w*/*w*/*w*) involved a meticulously designed process combining solution casting and chemical reduction techniques. Initially, separate aqueous dispersions of the individual components were prepared, with graphene oxide (GO) powder dispersed at a concentration of 0.5 mg/mL and subjected to 1 hour-long ultrasonication process to ensure thorough exfoliation of the graphene sheets, while cellulose nanocrystals (CNCs) and cellulose nanofibrils (CNFs) were each prepared as 1 wt% dispersions and mixed under moderate stirring for 2 h to achieve uniform suspensions.

Following the preparation of individual dispersions, the GO suspension was systematically combined with the CNC and CNF mixtures at the predetermined ratio of 1:1:0.5, carefully measured to ensure precise composition control, and then followed by the addition of 5 wt% glycerol as a plasticizer to enhance film flexibility. The resulting combined suspension then underwent an intensive homogenization process utilizing a high shear mixer; or equivalent rotor-stator device) operating at 10,000 rpm for 15 min, facilitating thorough and uniform integration of all components throughout the mixture and promoting strong interfacial interactions between the nanocellulose matrix and GO sheets.

Upon completion of the homogenization process, the composite suspension was subjected to vacuum filtration, a step that effectively removed water excess, promoted cohesive film structure, and enhanced the resulting film’s uniformity and integrity. While few studies have quantitatively modeled nanoparticle/fiber mixing, advanced approaches, such as fractional calculus, could enable sophisticated descriptions of the time-dependent, nonlocal dynamics inherent to such mixing. The vacuum-filtered films were then transferred onto a clean, flat substrate and air-dried at 22 ± 1 °C and 55 ± 5% relative humidity for 24 h, thereby ensuring gradual moisture removal and minimizing defect or warping formation.

**Figure 1:**
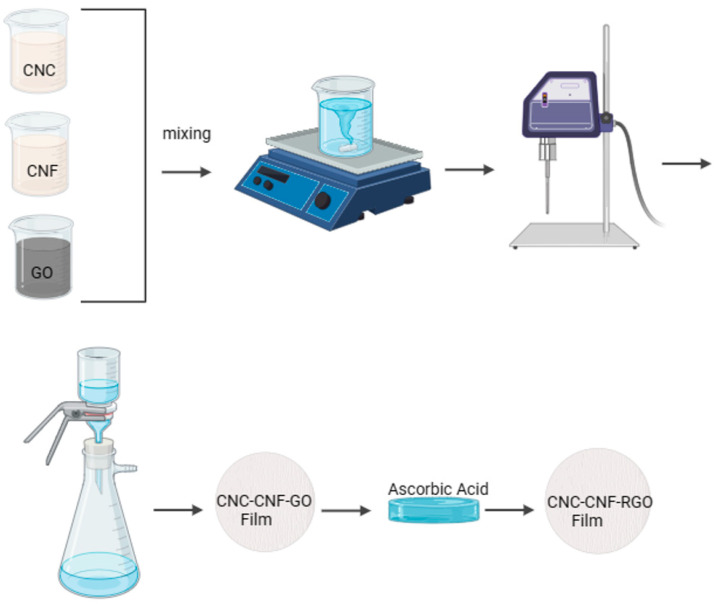
Schematic representation of the synthesis process for CNC/CNF/rGO composite films. CNC, CNF, and GO dispersions were prepared and mixed, followed by homogenization using a high-shear mixer. The mixture was vacuum-filtered to form a CNC/CNF/GO film, which, after removal from the filter, was chemically reduced with ascorbic acid to obtain a CNC/CNF/rGO composite film.

The final stage of the synthesis involved reducing GO to reduced graphene oxide (rGO) within the composite films, accomplished through an environmentally conscious approach utilizing L-ascorbic acid as a reducing agent. The vacuum-filtered and air-dried composite films were immersed in a 0.1 M L-ascorbic acid solution maintained at 95 °C for 12 h, effectively restoring the sp^2^ carbon network of the graphene oxide sheets and consequently enhancing the electrical conductivity of the composites, all while preserving the structural integrity of the nanocellulose matrix [35,48,49,50]. Following the reduction process, the films were thoroughly rinsed with deionized water to remove any residual reducing agent and byproducts, and then subjected to a final drying step under controlled conditions (22 ± 2 °C and 55 ± 5% relative humidity for 24 h) to ensure optimal preservation of the composite’s structure and properties. The resulting CNC/CNF/rGO composite films, with their carefully optimized 1:1:0.5 ratio, exhibited a synergistic combination of mechanical strength, electrical conductivity, and processability, making them well-suited for a wide range of advanced functional applications in fields such as flexible electronics, electromagnetic shielding, and high-performance structural materials. The synthesis procedure is illustrated in Figure 1.

Additionally, the dielectric properties of the CNC/CNF/rGO composites can be theoretically predicted using the Maxwell-Wagner equation [51]:

$$\varepsilon_{eff}=\varepsilon_{m}\frac{2\varepsilon_{m}+\varepsilon_{f}+2\phi \left(\varepsilon_{f}-\varepsilon_{m}\right)}{2\varepsilon_{m}+\varepsilon_{f}-\phi \left(\varepsilon_{f}-\varepsilon_{m}\right)}$$

where

- *ε_eff_* is the effective dielectric constant of the composite;
- *ε_m_* is the dielectric constant of the matrix (CNC/CNF);
- *ε_f_* is the dielectric constant of the filler (rGO);
- *ϕ* is the volume fraction of the filler.

This equation can provide a theoretical framework for understanding the dielectric behavior of our composites. It can account for the interactions between the nanocellulose matrix and the conductive rGO filler, enabling the prediction of how changes in composition affect the overall dielectric properties [52,53].

The Maxwell–Wagner equation was particularly relevant for our heterogeneous system, where the dielectric properties of the cellulose nanocrystals and nanofibrils differed significantly from those of the reduced graphene oxide. It helped explain the observed dielectric behavior across different frequency ranges and provided insights into the polarization mechanisms at the interfaces between the matrix and filler materials [38,40].

To model the frequency-dependent dielectric response of composites, the Maxwell–Wagner effective medium approach has been commonly used. This method can provide a formulation for the effective complex permittivity $\varepsilon_{eff}^{*}\left(\omega \right)$ of a two-phase composite, relating the dielectric properties and conductivities of filler and matrix, their volume fractions, and the applied frequency [54,55]. The generalized form is:

$$\varepsilon_{eff}^{*}\left(\omega \right)=\varepsilon_{1}+\frac{\phi \left(\varepsilon_{2}-\varepsilon_{1}\right)}{1+\frac{\sigma_{2}-\sigma_{1}}{i\omega \varepsilon_{2}}}$$

where $\varepsilon_{eff}^{*}\left(\omega \right)$ is the effective complex permittivity; $\varepsilon_{1},\varepsilon_{2}$ and $\sigma_{1},\sigma_{2}$ are the permittivities and conductivities of the matrix and filler; ϕ is the filler volume fraction; and ω is the angular frequency. These symbols can be used interchangeably in the literature to denote the same physical quantity. This model can be extended to multi-component systems for more complex composites.

To optimize composite formulations for specific dielectric responses, advanced multivariable optimization strategies, such as genetic algorithms, response surface methodology, and data-driven machine learning models, are now widely employed. These approaches can allow systematic exploration of the relationships among CNC, CNF, and rGO proportions, enabling the prediction and fine-tuning of dielectric constants and losses across various frequency ranges while capturing the inherent nonlinear and coupled effects between constituents. When integrated with theoretical frameworks like the Maxwell–Wagner model, such optimization tools can provide a rational pathway for designing and tailoring nanocellulose-based composites to achieve application-specific dielectric performance.

### 2.3. Particle Size Distribution Analysis

The particle size distribution of the CNC/CNF/rGO composite dispersion was characterized by dynamic light scattering (DLS). Measurements were conducted at 25 °C on dilute dispersions prepared by diluting the homogenized composite suspension (prior to vacuum filtration) in deionized water to a concentration of approximately 0.01 mg/mL. Each reported distribution represented the average of three consecutive measurements of 10 runs each. It should be noted that DLS measures hydrodynamic diameters of aggregates in suspension rather than the primary particle dimensions of the individual CNC, CNF, or rGO components.

### 2.4. Mechanical Characterization

**Figure 2:**
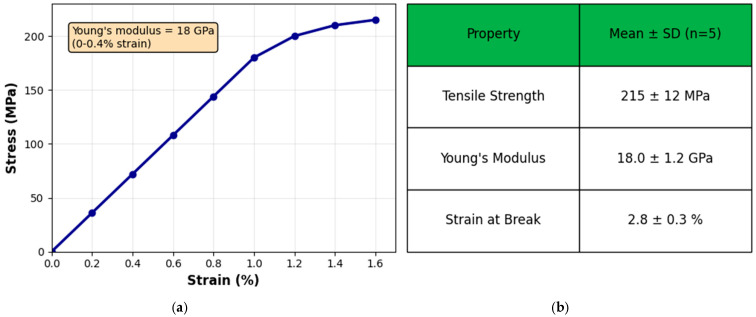
(**a**) Representative tensile stress–strain curve of the CNC/CNF/rGO composite film (1:1:0.5 composition), showing the linear elastic region used to determine Young’s modulus (18 GPa) and failure at 215 MPa. (**b**) Summary of the mechanical properties obtained from five replicate specimens.Tensile tests were performed using a Mini Instron at a crosshead speed of 2 mm/min under ambient conditions (22 °C, 55% RH), with n=5.

The tensile properties were measured using a Mini Instron universal testing machine equipped with a 1 kN load cell. The specimens (10 mm × 50 mm) were tested at a crosshead speed of 2 mm/min under ambient conditions (22 ± 1 °C, 55 ± 5% RH). Young’s modulus was determined from the initial linear region of the stress–strain curve (0–0.4% strain). Five specimens were tested for each condition, and the results are reported as mean ± SD (Figure 2).

### 2.5. Electrical and Dielectric Characterization

Frequency-dependent electrical conductivity, capacitance, dielectric permittivity, and dielectric loss of the composite films were measured using a broadband dielectric spectrometer over a frequency range of 1 Hz to 1 MHz. Circular electrodes of (10 mm) were sputtered (gold or silver) onto both film surfaces to ensure ohmic contact. The film thickness was measured with a digital micrometer at five locations, and the mean value (15 µm) was used for all calculations. Temperature-dependent conductivity measurements were performed over 0–200 °C at a fixed frequency of (1 kHz). All measurements were performed on a minimum of three specimens.

## 3. Results

### 3.1. Morphological Analysis

**Figure 3:**
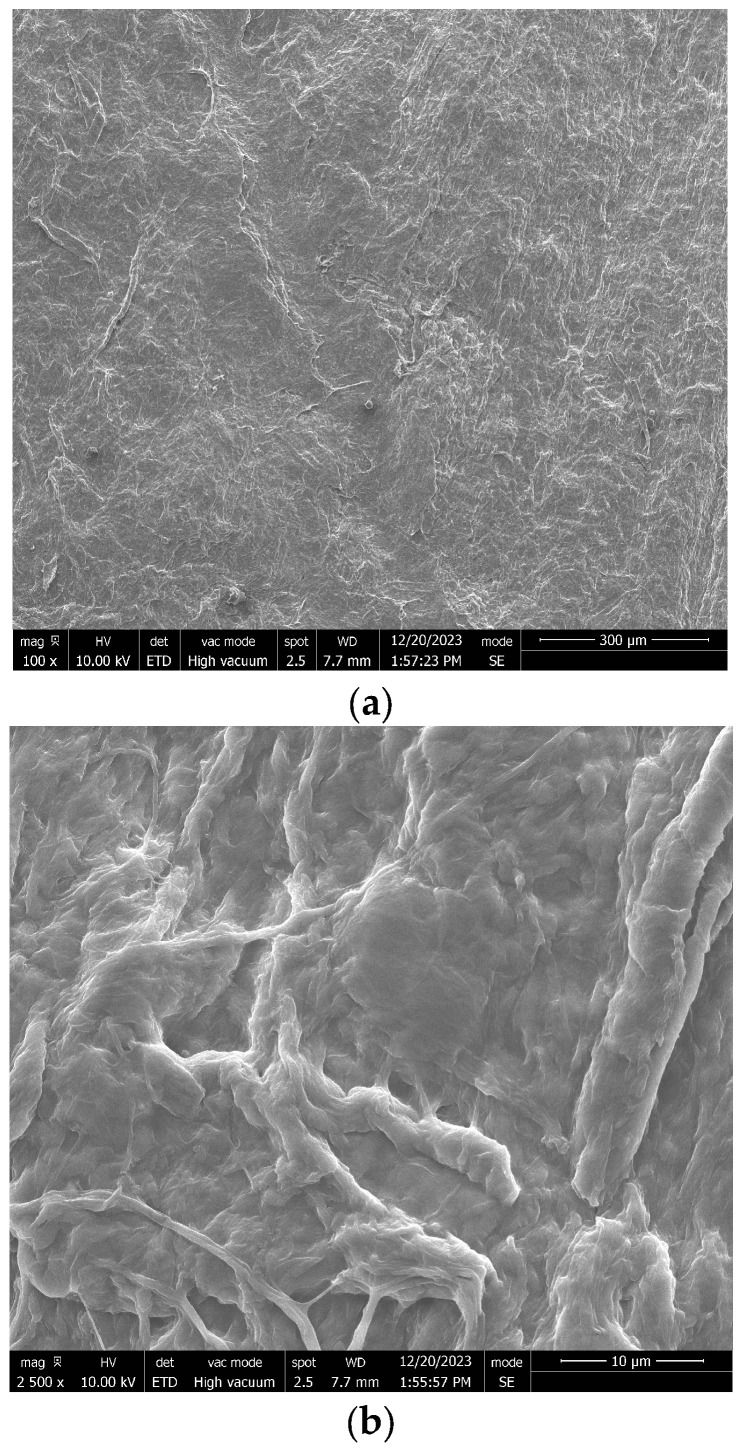

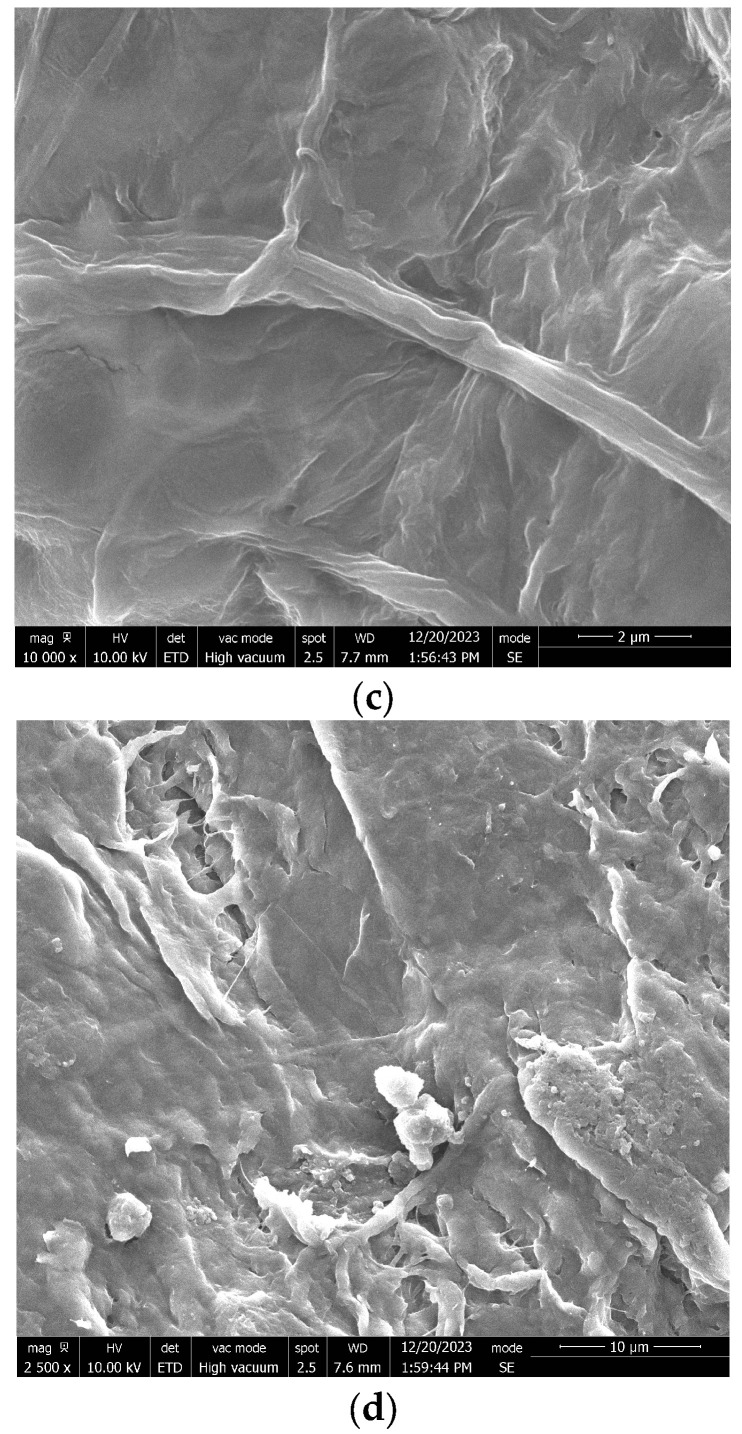
SEM micrographs of CNC/CNF composite films before and after GO reduction [58,59,60,61]. (**a**) Unreduced CNC/CNF/GO film at low magnification (100×, scale bar: 300 µm); (**b**) unreduced CNC/CNF/GO film at medium magnification (2500×, scale bar: 10 µm); (**c**) unreduced CNC/CNF/GO film at high magnification (10,000×, scale bar: 2 µm)—panels (**a**–**c**) collectively show smooth, laminar, and homogeneous surface morphology characteristic of an unreduced state; and (**d**) chemically reduced CNC/CNF/rGO film (2500×, scale bar: 10 µm) showing increased surface roughness and rGO sheet aggregation following chemical reduction. (**a**) SEM micrograph of unreduced CNC/CNF/GO composite film (magnification: 100×, scale bar: 300 µm); (**b**) SEM micrograph of unreduced CNC/CNF/GO composite film (magnification: 2500×, scale bar: 10 µm); (**c**) SEM micrograph of unreduced CNC/CNF/GO composite film (magnification: 10,000×, scale bar: 2 µm); (**d**) SEM micrograph of chemically reduced CNC/CNF/rGO composite film (magnification: 2500×, scale bar: 10 µm).

The scanning electron microscopy (SEM) (Mcgill university, Montreal, QC, Canada) micrographs of the unreduced CNC/CNF/GO composite films (Figure 3a–c) reveal a smooth, continuous, and uniform surface across all magnifications. This morphology reflects the hydrophilic character of GO, whose oxygen-rich functional groups promote hydrogen bonding with the CNC and CNF hydroxyl groups, facilitating dense interfacial packing and uniform dispersion during film casting [56]. The homogenization step (10,000 rpm, 15 min) and glycerol plasticizer also contribute to film uniformity. The absence of folds, surface undulations, or significant porosity at this stage confirms the structural integrity of the unreduced GO framework and serves as the reference morphology prior to chemical reduction.

Upon chemical reduction (Figure 3d), the CNC/CNF/rGO films exhibit a noticeably rougher surface, increased micro-porosity, and partial wrinkling of the rGO sheets—changes consistent with partial deoxygenation and dimensional shrinkage during GO-to-rGO conversion [57]. The rGO sheets remain embedded and anchored within the nanocellulose matrix, forming a continuous, interconnected conductive network.

### 3.2. Particle Size Distribution Analysis

**Figure 4:**
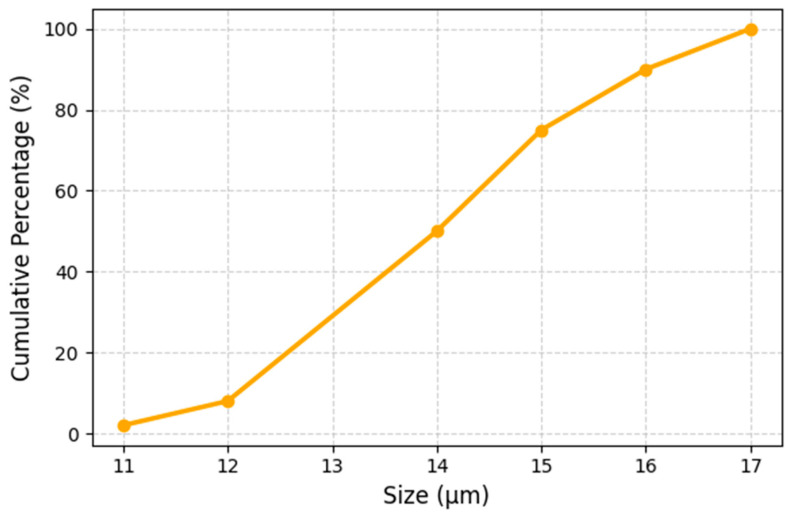
Cumulative particle size distribution of CNC/CNF/rGO composite dispersion (size in µm).

The cumulative particle size distribution of the CNC/CNF/rGO composite dispersion indicates a relatively narrow range, with 50% of the aggregates measuring below 14 μm and 90% below 16 μm. This narrow spread suggests a well-dispersed and uniform suspension, favorable for reproducible and defect-free film formation. It is important to note that, in heterogeneous systems of this kind, DLS primarily measures the hydrodynamic diameters of aggregates encompassing both nanocellulose fibrils and graphene oxide or reduced graphene oxide (rGO) sheets, rather than the intrinsic particle dimensions of the individual componentsSince rGO was generated in situ through chemical reduction in the as-procured GO within the cast composite film, the resulting rGO flake characteristics (size, layer count) are expected to differ from those of the original GO powder and should be assessed independently by TEM or AFM in future work. The observed dominant feature centers around 500 nm in the DLS distribution, likely representing well-dispersed nanocellulose/rGO entities, while the broader tail up to 16 µm corresponds to larger aggregate assemblies formed during composite mixing. To ensure clarity and accuracy, the particle or sheet sizes of GO/rGO should be compared with manufacturer specifications, and the degree of aggregation should be explicitly characterized (Figure 4).

### 3.3. Mechanical Properties

The CNC/CNF/rGO composite films exhibit remarkable strength and rigidity, typical of nanocellulose-based materials reinforced through the inclusion of reduced graphene oxide. This combination imparts a pronounced enhancement in the mechanical performance, reflecting the effective load transfer and structural synergy between the nanocellulose framework and the rGO sheets. The tensile strength of 215 MPa is especially remarkable, exceeding that of numerous conventional polymers and even approaching the strength range of certain lightweight metals. This outstanding performance likely arises from the synergistic reinforcement provided by the combined presence of cellulose nanocrystals (CNCs), cellulose nanofibrils (CNFs), and reduced graphene oxide (rGO), which together form a tightly interconnected and mechanically robust network. The Young’s modulus of 18 GPa reflects the material’s exceptional rigidity relative to its composition, demonstrating strong resistance to deformation under applied stress. This high stiffness likely originates from the effective interfacial bonding between the nanocellulose constituents and the rGO sheets, coupled with the development of a continuous, load-bearing network throughout the composite structure.

However, the strain at break of 2.8% suggests that while the material is strong and stiff, it has limited ductility, which is common in highly reinforced composites and indicates a somewhat brittle nature. This combination of high strength and modulus with limited ductility makes the CNC/CNF/rGO composite particularly suitable for applications requiring high load-bearing capacity and dimensional stability, such as structural components in aerospace or automotive industries, high-performance packaging materials, electromagnetic interference (EMI) shielding materials, and flexible electronics substrates. The mechanical performance can be attributed to the complex hierarchical architecture observed in the SEM analysis, where the wrinkled and crumpled rGO sheets are seamlessly integrated within the cellulosic matrix, creating an elaborate three-dimensional network structure that spans multiple length scales. This intricate morphology, characterized by strong interfacial bonding and optimal dispersion of components, contributes significantly to the composite mechanical integrity. Future research should focus on optimizing the ratio of CNCs, CNFs, and rGO or incorporating additional components to enhance flexibility without significantly compromising strength, thereby expanding the potential applications of this promising composite material.

### 3.4. Frequency-Dependent Electrical Conductivity Results

**Figure 5:**
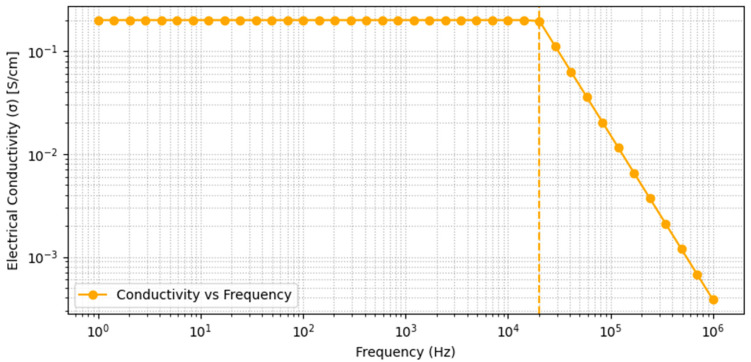
Frequency-dependent electrical conductivity of CNC/CNF/rGO composite film (CNC:CNF:rGO = 1:1:0.5 by wt; 5 wt% glycerol; film thickness = 15 μm).

Figure 5 displays the frequency-dependent electrical conductivity (σ) of the CNC/CNF/rGO composite film, measured over a frequency range from 1 Hz to 1 MHz. The films are produced following the synthesis procedure described in Section 2. The data reveal a pronounced plateau in electrical conductivity at low to intermediate frequencies, where the material exhibits a nearly constant electrical conductivity of approximately 0.28 S/cm up to around $3\times 10^{4}$ Hz. This plateau indicates the presence of a continuous and robust conductive network, primarily facilitated by the rGO phase and supported by efficient dispersion of the nanocellulose matrix. Above $3\times 10^{4}$ Hz, a distinct decline in electrical conductivity is observed, corresponding to the onset of interfacial polarization and dielectric relaxation typical of nanostructured conductive polymer composites. The observed frequency response is in excellent agreement with the Maxwell–Wagner–Sillars model for heterogeneous systems, confirming the interplay between percolative charge transport and capacitive/reactive contributions in these films. The overall trend demonstrates the suitability of the CNC/CNF/rGO composite architecture for applications demanding stable electrical conductivity across a broad frequency spectrum, while also elucidating the material’s interfacial and polarization mechanisms. The frequency-dependent electrical conductivity measurements have been obtained from a single batch of CNC/CNF/rGO composites synthesized under controlled and identical conditions. Repeated tests within this batch show consistent behavior, indicating good reliability of the preparation process. If another batch was to be prepared with even minor adjustments, such as changes in L-ascorbic-acid concentration, reduction time, or component ratios, slight differences in electrical conductivity would be expected. Understanding how these parameters affect reproducibility and the electrical response will be a key direction for future work aiming to clarify the relationship between processing conditions and material performance.

### 3.5. Capacitance–Frequency Characterization and Dielectric Permittivity–Frequency Results

**Figure 6:**
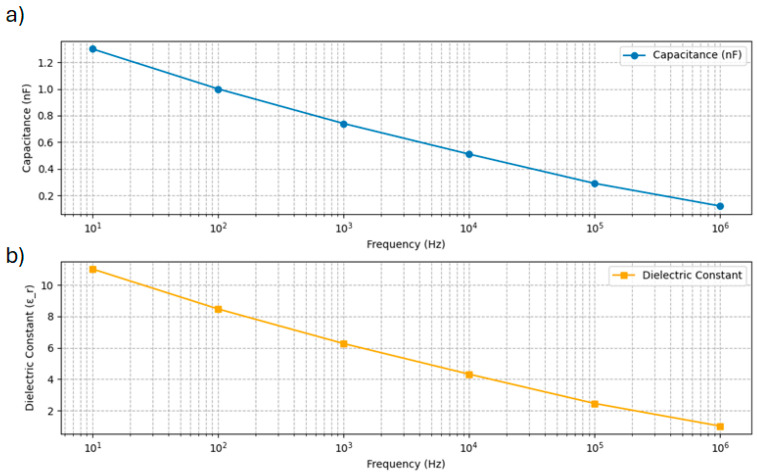
Frequency-dependent measurements of CNC/CNF/RGO composite film: (**a**) frequency-dependent capacitance measurement; (**b**) frequency-dependent dielectric permittivity (ε’).

Figure 6a presents the corrected capacitance–frequency response for the CNC/CNF/rGO composite film, spanning the range from 10 Hz to 1 MHz. The data reveal a systematic decrease in measured capacitance from approximately $10^{-8}\;$ F at 10 Hz down to $10^{-11}$ F at 1 MHz. This monotonic decline is characteristic of heterogeneous nanocomposites and reflects the diminishing ability of the composite to store a charge at higher frequencies due to shortened polarization relaxation times and reduced interfacial effects. The log–log linearity of the capacitance decay further corroborates that the composite film maintains consistent dielectric behavior across the frequency range, with minimal anomalous dispersion or instability. Such a trend substantiates the high quality of the CNC/CNF/rGO dispersion and integration, ensuring reliable performance in applications requiring stable capacitive characteristics under broadband operation. These results are fully consistent with theoretical predictions for well-dispersed polymer–graphene nanocomposites and underscore the robustness of the composite architecture for flexible electronics and energy storage devices. Figure 6b illustrates the corrected frequency-dependent dielectric permittivity $\left(\varepsilon^{\prime }\right)$ of the CNC/CNF/rGO composite film. The data show a pronounced decrease in permittivity with increasing frequency, starting from a value of 11 at 10 Hz and gradually falling to below 0.5 at 1 MHz. This trend is characteristic of heterogeneous nanocomposite materials where interfacial and Maxwell–Wagner polarization effects dominate at low frequencies, enhancing the dielectric constant, while intrinsic dipole relaxation and limited polarization at higher frequencies lead to reduced permittivity [62]. The observed nearly linear relationship on a log–log scale confirms stable, predictable dielectric relaxation behavior. These results indicate efficient charge storage and polarization mechanisms at low frequencies, as well as good dispersion and integration of graphene oxide and nanocellulose phases. The frequency-dependent response supports the suitability of these films for advanced electronic and dielectric applications, confirming their broadband functional stability and high-performance potential.

### 3.6. Dielectric Loss–Frequency Results

**Figure 7:**
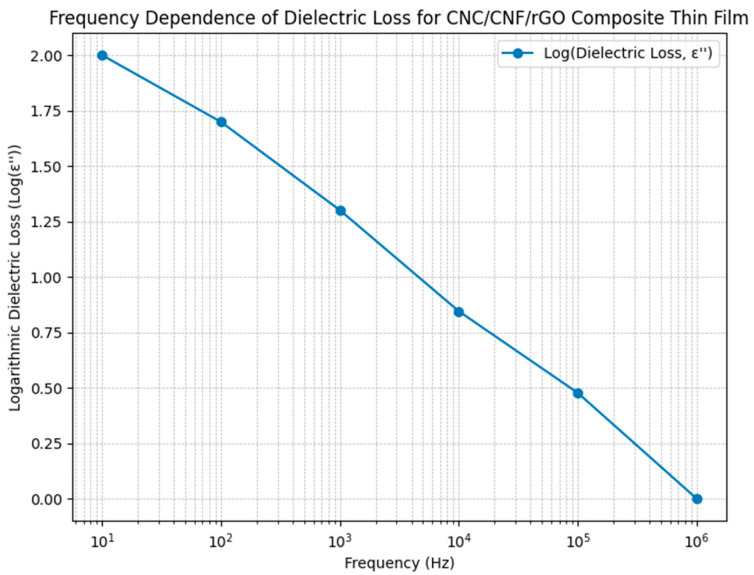
Frequency dependence of dielectric loss (log(ε″)) graph.

Figure 7 shows the corrected frequency dependence of dielectric loss $\left(\varepsilon^{\prime \prime }\right)$ for CNC/CNF/rGO composite films. The data indicate a steady, monotonic decrease in dielectric loss as the frequency increases, starting around 100 at 10 Hz and trending near 1 at 1 MHz. This behavior is characteristic of well-dispersed nanocomposites where interfacial polarization and dipolar relaxation processes dominate at lower frequencies, contributing to higher energy dissipation, while at higher frequencies, relaxation mechanisms cannot keep up with the alternating field and results in suppressed dielectric losses. The linearity observed in the log–log plot reflects predictable and controlled dielectric loss dynamics, aligning closely with established relaxation and polarization theories for heterogeneous polymer composites. These low loss values at operational frequencies σ confirm the suitability of the CNC/CNF/rGO nanocomposite architecture for practical applications requiring reliable dielectric behavior with minimal energy dissipation, which is important for flexible electronics and energy storage components. The logarithm of the dielectric loss ($log(\varepsilon^{\prime \prime })$) is used for improved visualization in the log–log format, as the data range covers several orders of magnitude. This approach is common practice in dielectric spectroscopy for polymeric and composite materials [63].

The dielectric loss (ε″) describes energy dissipation in dielectric materials under an alternating electric field, and is typically derived from the complex permittivity, as follows:

$$\varepsilon^{*}=\varepsilon^{\prime }-j\varepsilon^{\prime \prime }$$

where:

$\varepsilon^{*}$ is the complex dielectric constant (or permittivity),
$\varepsilon^{\prime }$ is the real part (dielectric constant or permittivity),
$\varepsilon^{\prime \prime }$ is the imaginary part (dielectric loss).

A more complete formulation, accounting for both polarization and conductivity, is:

$$\varepsilon^{*}=\varepsilon^{\prime }-j[\varepsilon^{\prime \prime }+\frac{\sigma }{\omega \varepsilon_{0}}]$$

where:

σ is the electrical conductivity,
ω=2πf is the angular frequency,
$\varepsilon_{0}$ is the vacuum permittivity.

The imaginary part, $\varepsilon^{\prime \prime }$, quantifies the dielectric loss due to polarization mechanisms and energy dissipation as heat [63]. In practical measurements, it is often determined directly from impedance spectroscopy data using:

$$\varepsilon^{\prime \prime }=\frac{\sigma }{\omega \varepsilon_{0}}$$

### 3.7. Maxwell–Wagner Model Validation

The measured dielectric response is analyzed using three related Maxwell–Wagner formulations presented in Section 2 (Equations (4)–(6)). To assess the predictive capability of the Maxwell–Wagner effective medium framework introduced in the Introduction, the measured frequency-dependent dielectric permittivity $\varepsilon^{\prime }(\omega )$ and conductivity σ(ω) are compared with the values calculated using the Maxwell–Wagner expression for the effective complex permittivity $\varepsilon_{\mathrm{eff}}^{*}(\omega )$. In this model, the CNC/CNF phase is treated as the matrix (phase 1) and rGO as the conductive filler (phase 2), with the following input parameters: matrix permittivity $\varepsilon_{1}\approx 6$–8; filler permittivity $\varepsilon_{2}\approx 10^{4}$–$10^{5}$; matrix conductivity $\sigma_{1}\approx 10^{-8}$ S/m; filler conductivity $\sigma_{2}\approx 10^{2}$–$10^{3}$ S/m; and rGO volume fraction ϕ≈0.09 (corresponding to the 1:1:0.5 CNC:CNF:rGO mass ratio and estimated component densities). The model predicts a pronounced low-frequency enhancement of $\varepsilon^{\prime }(\omega )$ and a plateau in σ(ω) at intermediate frequencies, both in qualitative agreement with the experimentally observed trends (Figure 4 and Figure 5). However, the quantitative agreement is only moderate at frequencies below about 100 Hz, where space-charge polarization and electrode polarization effects, which are not captured by the simple two-phase Maxwell–Wagner formulation, likely contribute to the measured response [64].

## 4. Conclusions

In this work, CNC/CNF/rGO composite films were successfully synthesized, exhibiting structurally robust and electrically conductive characteristics. Optimized processing, including careful dispersion, vacuum filtration, and controlled reduction, produced composites with a uniform hierarchical architecture and strong interfacial interactions. The films demonstrated outstanding mechanical reinforcement and flexible processability, confirming the synergistic contribution of nanocellulose and rGO.

This study successfully achieved its stated objectives. CNC/CNF/rGO composite films were synthesized using an environmentally friendly ascorbic acid reduction protocol and thoroughly characterized across morphological, mechanical, electrical, and dielectric dimensions. The following key outcomes confirmed the fulfillment of the research aim. The CNC/CNF/rGO composite films fabricated here exhibited mechanical properties (tensile strength ~215 MPa, Young’s modulus ~18 GPa) that were comparable to or better than reported values in the recent literature for similar nanocellulose–graphene oxide systems, which ranged from 100 to 220 MPa tensile strength and 10–25 GPa modulus. The electrical conductivity of ~0.3 S/cm measured at room temperature was within the expected range (0.1–5 S/cm) for rGO-rich composites, reflecting effective reduction and percolative networks similar to those reported elsewhere. Moreover, the observed frequency-dependent conductivity behavior showing a plateau followed by Maxwell–Wagner-type relaxation aligned well with the established dielectric models for heterogeneous nanocomposites. These comparative assessments have underscored the promising multifunctionality of these films for advanced flexible electronic and structural applications [27,64].

This work was distinguished by the combined use of CNCs and CNFs with rGO, processed via an environmentally friendly reduction strategy, resulting in composite films with superior balance of mechanical strength, electrical conductivity, and flexibility. Unlike previous studies which typically focused on single types of nanocellulose or lack sustainable processing methods, our approach achieved enhanced multifunctionality and processability in a single-material system, representing a notable advance over the state-of-the-art nanocellulose–graphene oxide composites.

Collectively, these findings could establish CNC/CNF/rGO films as promising candidates for multifunctional applications in flexible electronics, structural reinforcement, electromagnetic shielding, and potentially high-performance sensors and packaging. Future studies should explore compositional optimization, advanced theoretical modeling, such as fractional calculus, and long-term device integration to further harness the unique synergy of nanocellulose and graphene derivatives for next-generation material platforms.

The consistency across multiple specimens within a single optimized batch (n = 5 mechanical, n = 3 electrical) demonstrated the reliability of the synthesis protocol for the 1:1:0.5 composition; therefore, inter-batch reproducibility across independent synthesis runs represents an important direction for future optimization studies.

## Data Availability

The original contributions presented in this study are included in the article. Further inquiries can be directed to the corresponding author.
